# Clinical Management of Testicular Tumors in Dogs

**DOI:** 10.3390/ani16081202

**Published:** 2026-04-15

**Authors:** Maria Pereira, Koray Tekin, Malena Perez, Kurt de Cramer, Stefano Romagnoli

**Affiliations:** 1 Department of Animal Medicine, Production and Health, University of Padova, 35020 Padova, Italy; maria.pereira@unipd.it (M.P.); malena.perez@studenti.unipd.it (M.P.); stefano.romagnoli@unipd.it (S.R.); 2 Department of Reproduction and Artificial Insemination, Faculty of Veterinary Medicine, Ankara University, Ankara 06110, Türkiye; 3 Rant en Dal Animal Hospital, Johannesburg 1751, South Africa; kdcramer@mweb.co.za; 4 Department of Production Animals, Faculty of Veterinary Science, University of Pretoria, Pretoria 0110, South Africa

**Keywords:** canine testicular tumors, hyperestrogenism, Leydig cell tumor, preputial cytology, seminoma, Sertoli cell tumor

## Abstract

Testicular tumors are the most common neoplasms of the canine male reproductive tract, corresponding to approximately 25% of all tumors in intact males. A large percentage of cases are characterized by one of three main tumor types: seminomas, interstitial Leydig cell tumors, or Sertoli cell tumors. Clinical importance is primarily associated with endocrine activity rather than malignant behavior; orchiectomy is the treatment of choice for most canine testicular cancers. Endocrine activity, particularly estrogen secretion, may result in feminization syndrome and, in severe cases, bone marrow suppression. The diagnostic approach combines physical examination, ultrasonography with hormonal assessment using endocrine testing (testosterone, estradiol, and T:E ratio), and/or tissue level evidence of the estrogen effect (preputial cytology). Management is centered on orchiectomy; unilateral surgery may be considered when the contralateral testis is clinically and ultrasonographically normal and when preservation of reproductive capacity or working ability is still a priority. Dogs with hormonally active tumors benefit from postoperative hematologic and endocrine monitoring. Recent advances in immunohistochemistry (IHC), such as Ki-67 and inhibin-α markers, and imaging techniques are improving tumor characterization and individualized clinical decision making.

## Simple Summary

Testicular tumors are common in intact, older male dogs. Whilst the majority of these tumors are benign, some are malignant and may metastasize. In addition, some testicular tumors are hormonally active and may have systemic effects. Estrogen is the most common offending and clinically significant hormone produced by testicular tumors, leading to feminization syndrome and sometimes bone marrow suppression that disrupts normal blood cell production. In some cases, excessive androgen production may lead to benign prostatic hypertrophy, which may ultimately progress to prostatitis, cyst formation and abscessation. The manuscript emphasizes the importance of routine reproductive health monitoring in intact older dogs to ensure early detection. Surgical removal of the affected testicle is usually curative and may be best practice for some dogs with a testicular tumor in only one testicle, whilst in others bilateral orchiectomy (removal of both testicles) may be indicated. Increased vigilance and knowledge on testicular tumors improve animal welfare, support informed choices for owners, and reduce the chance of severe complications in older male dogs.

## 1. Introduction

Testicular tumors are the most common neoplasms of the canine male reproductive tract, representing 16–27% of tumors in intact male dogs and up to 90% of all neoplasms of the male genital system [1,2,3,4,5,6,7]. Across populations, they account for roughly 1–2% of all canine cancers, and the prevalence can exceed 20% in geriatric intact males. In a population-based study evaluating tumors in both sexes, testicular tumors represented 2.35% of the 67,943 recorded neoplasms [8].

Most canine testicular tumors are diagnosed in middle-aged to geriatric animals, with a mean age at diagnosis of around ten years [3,7,9]. The three major histological types are Sertoli cell tumors (SCTs), Leydig cell tumors, also known as interstitial cell tumors (ICTs), and seminomas (SEMs), collectively comprising over 95% of canine testicular neoplasms [3,4,10]. The remaining tumors reported are rare mixed tumors and poorly differentiated neoplasms [11,12].

Most testicular tumors are non-invasive and exhibit low metastatic potential [3,4,12], especially ICTs [13]. However, apart from structural impact, SCTs and rarely ICTs may produce estrogen and excessive androgen levels, respectively leading to feminization syndrome [10,14] and androgen-related pathology such as prostate pathology, perianal gland tumors and perineal hernias. Confirmation of tumor type is not predictive of hormone production nor hormone type. Approximately 25–30% of SCTs are hormonally active and produce estrogens, resulting in classical feminization syndrome and, in selected cases, bone marrow suppression. Despite their endocrine impact, true malignant behavior remains uncommon, with metastases reported in fewer than 15% of cases and typically involving the inguinal or sub-lumbar lymph nodes [4,10]. In addition, in very rare instances ICTs may produce estrogen. Hormonally active tumors may have fatal paraneoplastic sequelae. Irreversible bone marrow suppression (myelotoxicity) may result from excessive estrogen production whilst excessive androgen production may lead to benign prostatic hypertrophy (BPH), which may ultimately progress to prostatitis, cyst formation, abscessation and death [15,16,17,18]. Early detection of testicular tumors is therefore crucial to allow for timeous medical or surgical intervention, thereby preventing potential mortality. Suspicion of testicular tumors may be raised by the physical examination and ultrasonography of the genital system. Endocrine assay (testosterone, estradiol, T:E ratio) aids in identifying those testicular tumors that produce hormones [18]. Preputial cytology is a practical inexpensive tool to help identify those tumors that produce estrogen [19,20,21]. The definitive standards for diagnosis and tumor classification are histopathology and immunochemistry (IHC) [3]. This manuscript aims to formulate the best current practice in the clinical management of testicular tumors in male dogs whether intended for breeding or not.

## 2. Epidemiology and Pathophysiology

The distribution of canine testicular tumors varies considerably across studies and appears to be influenced by population demographics and diagnostic criteria [3,4,7,22]. While SCT, ICT, and SEM types dominate, their relative frequency is inconsistent. Johnston et al. (2001) reported SCTs as the most prevalent [23], whereas more recent surveys suggest a relative shift toward a higher proportion of SEMs or ICTs [3,10,22]. The complexity of canine testicular neoplasia is highlighted by the fact that more than one type of tumor can be diagnosed in the same individual, with Nascimento et al. reporting this in 15.8% of cases [10]. This apparent change is unlikely to reflect a single underlying cause. Instead, it probably results from a combination of factors, including capture (necropsy vs clinical), increasing canine longevity, breed predisposition and a variation in how strictly tumors are classified histologically [7,24].

Age remains by far the most important risk factor for testicular tumor development in dogs. The incidence rises sharply after middle age, a trend that likely reflects the cumulative effect of time (progressive structural and cellular degeneration) with declining local immune control within testis and testicular degeneration of the germ and stromal cells [25,26]. In dogs with both scrotal testicles, the incidence of testicular tumors increases after 7–8 years [2,3,7,24]. The effect of age as a risk factor is further demonstrated by the observation that dogs belonging to long-lived breeds show disproportionally higher tumor rates than those with a shorter lifespan [8,10,24]. Cryptorchidism is a biological risk factor for testicular tumors that seems to be independent of age. Cryptorchid dogs may develop testicular tumors as early as 10 months of age [10] and cryptorchid dogs diagnosed later in life also show disproportionally higher tumor rates than age-matched dogs with two scrotal testes [2,24].

Interestingly, cryptorchidism also alters the risk for tumor type. Undescended testes are mainly SCTs and SEMs, while ICTs are almost exclusively found in scrotal testes together with SCTs and SEMs [9,27]. The elevated intra-abdominal temperature in retained gonads causes degeneration of germ and supporting cells, possibly explaining the increased susceptibility to neoplastic transformation [9] and possibly also tumor development at a young age. The right testis is more often retained, with its more cranial embryonic position and longer gubernacular path being suggested as playing a role [28].

Bilateral testicular tumors are reported in roughly 10–15% of affected dogs [4,7]. Metastatic disease has been documented in up to 15% of SCTs and SEMs, whereas ICTs rarely develop metastases [4,10,13].

Breed predisposition has been suggested, particularly for medium to large breeds such as Boxer, Golden Retriever, German Shepherd, Shetland Sheepdog, Collie, Weimaraner and Crossbreeds; however, no consensus has been reached for such associations [2,7,24]. Irrespective of breed, prompt clinical risk assessment is recommended. Cryptorchid dogs should be identified as early as is possible and the retained testicle removed if possible, and all intact male dogs should be examined for testicular health as they approach over five years of age as this represents the age at which scrotal testes may start developing tumors [9].

### 2.1. Types and Histopathological Features

Histopathological examination remains the definitive method for diagnosing and classifying canine testicular tumors. Initial histological assessment focuses on preservation (Supplementary Figure S1) or disruption of the normal seminiferous structure, growth pattern (intra-tubular vs. interstitial), and evidence of capsular penetration or stromal invasion [29].

Features that raise concern for aggressive biological behavior, regardless of tumor type, include marked cellular pleomorphism, high mitotic activity or atypical mitoses, vascular or lymphatic invasion, areas of necrosis or hemorrhage, extracapsular extension, and a clearly increased Ki-67 labeling index [30,31].

#### 2.1.1. Sertoli Cell Tumor

SCT arises from the Sertoli cells lining the seminiferous tubules, which play a central role in supporting germ cell development and maintaining the blood–testis barrier. Clinically, these tumors are typically slow-growing, firm and non-invasive, ranging from a few millimeters to several centimeters in diameter [4,27]. They occur both in scrotal and cryptorchid testes, but the incidence is significantly higher in retained gonads, particularly those located intra-abdominally [7,9,28,32,33,34].

**Figure 1:**
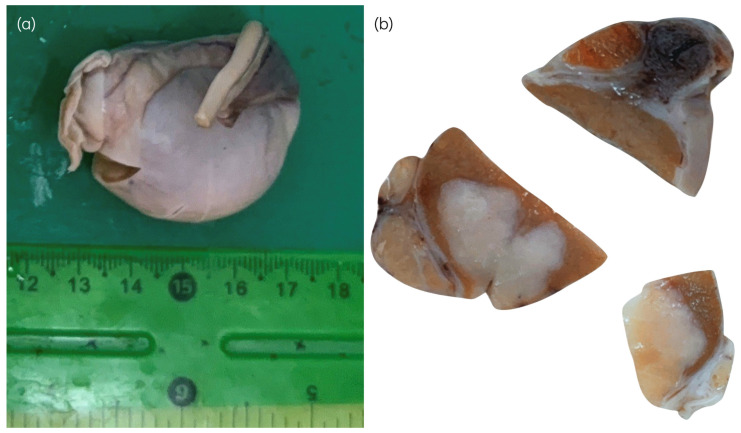
Macroscopic appearance of the testis from an 8-year-old male Bulldog. (**a**) Enlarged testis affected by a Sertoli cell tumor, measuring 5 × 3.5 × 3 cm, with a gray-white external appearance. (**b**) Cut section of the affected testis showing a solid, pale-gray to white mass with a firm and relatively homogeneous cut surface.

Macroscopically, affected testes often show a white to pale-grey color, firmness (Figure 1), and a lobulated mass, sometimes accompanied by cystic degeneration or focal hemorrhage [2,10,22]. Complete replacement of normal testicular parenchyma may occur in advanced cases, especially in long-standing cryptorchid tumors.

Histologically, SCTs display considerable architectural and cytological patterns. Well-differentiated forms are characterized by tubular or intra-tubular arrangements of elongated, columnar Sertoli cells, often separated by prominent fibrous stroma. In contrast, poorly differentiated types lose their tubular organization and consist of diffuse sheets or cords of polygonal cells with abundant cytoplasm, cytoplasmic vacuolization and prominent nucleoli (Supplementary Figure S2) [4,10]. Cytoplasmic vacuolization is a frequent and diagnostically useful feature, particularly in hormonally active tumors, and reflects intracellular accumulation of steroid lipids with estrogen synthesis [10,11]. Mitotic figures and nuclear pleomorphism may be present but do not, on their own, reliably predict malignant behavior [4].

#### 2.1.2. Interstitial (Leydig) Cell Tumors

**Figure 2:**
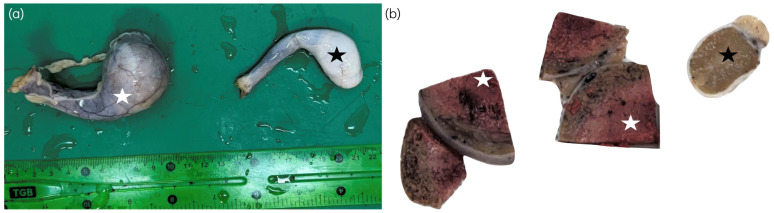
Macroscopic appearance of the testes from a 13-year-old male Beagle. (**a**) Enlarged left testis affected by an interstitial (Leydig) cell tumor (white star), measuring 5 × 3.5 × 3 cm, shown together with the contralateral right testis of normal size (black star). (**b**) Section of the testes with abnormal (white star) or normal (black star) testicular parenchyma.

ICTs, also referred to as Leydig cell tumors, originate from androgen-producing interstitial cells located between seminiferous tubules, outside the blood–testis barrier. These tumors are typically benign, small (often <1.0 cm), and non-functional, and are frequently detected incidentally during routine clinical examination, imaging or necropsy [3,10,22]. Macroscopically ICTs are well-described, yellow to brown nodules that may contain blood-filled cystic spaces (Figure 2) but generally cause minimal distortion of surrounding testicular parenchyma [2,4,10,11]. In contrast to SCTs and SEMs, ICTs are almost exclusively confined to scrotal testes, a feature that helps distinguish them epidemiologically from other testicular neoplasms [3,22].

Histologically, ICTs are composed of large polygonal cells with abundant eosinophilic, granular cytoplasm and round centrally positioned nuclei. Intracytoplasmic vacuolation is a common finding, reflecting the steroidogenic nature of these cells (Supplementary Figure S3). This functional phenotype can be further supported by immunohistochemical expression of steroidogenic markers such as inhibin-α, calretinin and 3β-hydroxysteroid dehydrogenase [11,35].

Although ICTs are classically considered hormonally inactive, rare functional variants may occasionally secrete excessive testosterone (hyperandrogenism) or, less commonly, produce estrogens (hyperestrogenism), leading to feminization. Hyperandrogenic ICTs may present clinically with increased libido or androgen-driven behavioral changes. Prostatic enlargement has been reported in these dogs, and this may be caused by BPH, which is an expected feature of many aging intact dogs but may be aggravated by increased androgen levels and conversion to dihydrotestosterone (DHT) acting as a potent stimulator for cell proliferation and hypertrophy [15,36]. Estrogen-producing ICTs are extremely uncommon but have been reported and may mimic feminizing SCTs, causing alopecia, gynecomastia, preputial enlargement, contralateral testicular atrophy, and in some cases, bone marrow suppression [12,13,18,37]. For this reason, clinical interpretation should not rely solely on tumor type, and preputial cytology and endocrine evaluation should be considered whenever testicular mass is identified, rather than assuming that ICTs are uniformly hormonally inactive.

#### 2.1.3. Seminoma

SEMs arise from germinal (spermatogenic) cells within the seminiferous tubules and account for 25–35% of all canine testicular tumors [5,7,22]. They are most common in scrotal testes but can also occur in retained abdominal testes [9].

**Figure 3:**
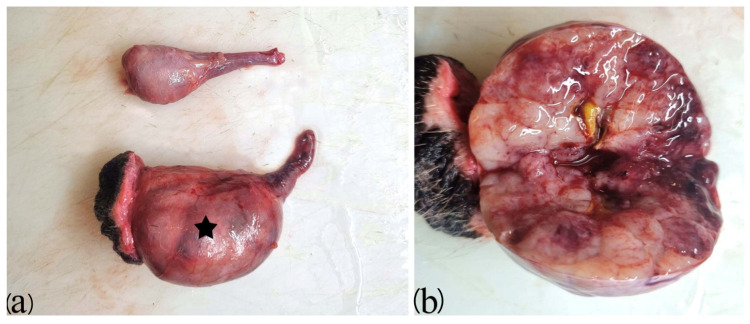
Macroscopic appearance of the testes from a 13-year-old male terrier. (**a**) Seminoma (star) and the contralateral testis of normal size. (**b**) Cut section of the testis diagnosed with seminoma (indicated by the star in part (**a**)), showing a multilobular appearance with white and reddish areas.

Grossly, SEMs present as soft, white to grey, lobulated masses that may replace the entire testis and often contain hemorrhagic or necrotic foci (Figure 3) [2,4,10,22]. Two histologic forms are recognized, diffuse and intra-tubular SEMs, in which neoplastic cells proliferate within the seminiferous tubules, where the tumor breaches the basement membrane and expands into the interstitium [4].

The SEM neoplastic cells are large and polyhedral, with eosinophilic cytoplasm, vesicular nuclei, and prominent nucleoli (Supplementary Figure S4). Mitotic figures are frequently observed, and lymphocytic infiltration may occur between neoplastic cells [10]. In early stages, SEMs are often seen next to seminiferous tubules that show atrophy or compression from the expanding masses. This spatial relationship supports the widely accepted concept that SEMs arise through a step-wise neoplastic transformation of spermatogonia [10].

Although most SEMs are benign, approximately 5% display invasive or metastatic behavior, usually spreading to regional lymph nodes [10]. Histological features such as vascular invasion, high mitotic index, pleomorphism and elevated Ki-67 labeling may suggest aggressive behavior, although reliable prediction of malignancy requires combined histopathological and clinical staging [4,38]. SEMs themselves lack intrinsic steroidogenic capacity [11]. Therefore, androgen excess observed in a subset of cases is presumed to arise from paracrine stimulation of adjacent Leydig cells within the tumor micro-environment, rather than direct hormone synthesis by neoplastic germ cells, a hypothesis supported by the frequent co-existence of SEM with ICT in the same testis, particularly the SEM-ICT combination identified in 44.4% of multiple tumor cases by Kuberka et al. (2025) [39]. Consequently, androgenic manifestation may occur in rare cases, whereas estrogen-related feminization remains implausible in pure SEM cases [10,11]. Surgical excision is curative, and postoperative prognosis is favorable.

## 3. Clinical Presentation

The clinical manifestations of canine testicular tumors depend largely on hormonal activity, size and location, which together determine whether the disease presents as a localized reproductive abnormality or a systemic endocrinopathy [4,10]. Case series and reports illustrate this spectrum well, ranging from asymptomatic dogs, infertility, scrotal enlargement, feminization, dermatologic changes, and prostatic disease [4,14]. A small subset of these patients may develop severe bone marrow suppression, pancytopenia and fatal consequences [14,40,41]. In addition, rare cases of metastatic ICTs with cutaneous or visceral involvement have been reported [13,14,42,43].

### 3.1. Local Clinical Signs

Most dogs are presented with asymmetric testicular enlargement when only one testis is involved, or rarely, bilateral involvement results in generalized scrotal enlargement. The affected gonad is usually firm and non-painful on palpation, distinguishing neoplastic enlargement from inflammatory conditions such as orchitis [4,10,23]. When tumors arise in retained abdominal or inguinal testes, abdominal distension or a palpable abdominal mass may be noted instead of scrotal asymmetry [9,22,27]. In some cases, the testicular enlargement is diffuse, while in others discrete nodules can be identified within an otherwise-normal parenchyma [11].

Large intra-testicular masses can cause pressure atrophy on seminiferous tubules of the contralateral testicle, leading to partial or complete spermatogenic failure. However, contralateral testicular atrophy is more commonly due to endocrine disruption rather than mechanical factors. In hormonally active (particularly estrogen-secreting) tumors, negative feedback on the hypothalamus–pituitary–gonadal (HPG) axis suppresses gonadotropin release, resulting in functional regression of the contralateral testis. Retained abdominal testes are predisposed to spermatic cord torsion, particularly when enlarged by neoplasia, which may present with acute abdominal pain, lethargy, vomiting, fever and abdominal distension [11,34,43].

### 3.2. Systemic and Endocrine-Related Signs

**Figure 4:**
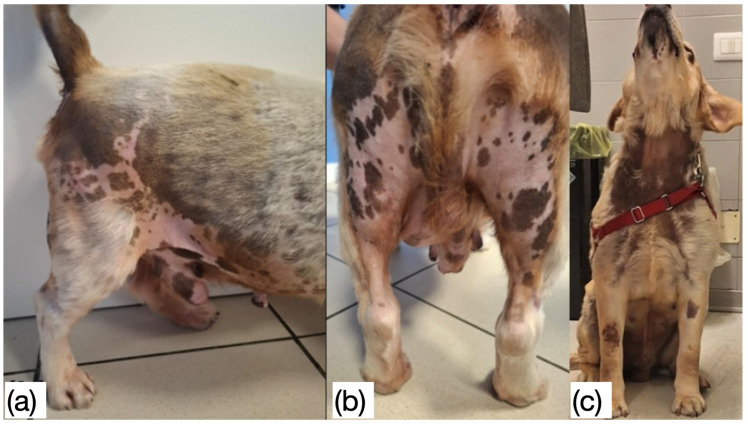
Systemic and endocrine related presentations of canine feminization syndrome associated with estrogen-producing testicular tumors [19]. Ten-year-old mixed-breed cryptorchid male dog with a Sertoli cell tumor. Notice the diffuse and symmetric alopecic areas in the flank, ventral abdomen, inguinal region and rear limbs (**a**), in addition to nipple enlargement and pendulous prepuce, and the presence of a left abdominal mass corresponding to the retained left testicle (**b**). The lesion patterns and distribution (non-pruritic, bilaterally symmetric hair with darkening of the skin) are typical of endocrine-related (generally hyperestrogenism) alopecia (**c**).

Systemic alterations occur predominantly in hormonally active tumors, especially estrogen-secreting SCTs. Sertoli cells and, to a lesser degree, Leydig cells possess the enzymatic machinery for aromatization of testosterone and androstenedione into 17 β-estradiol and estrone [11]. Tumors derived from these cells may induce a feminization syndrome. Common clinical features include dermatological alterations such as non-pruritic, bilaterally symmetrical alopecia beginning in the genital or perineal region and extending to the flanks, thorax, and neck, along with hyperpigmentation and skin thinning [3,44]. Reproductive changes include gynecomastia, galactorrhea, and enlargement of the prepuce with decreased penile tone [45]. Behaviorally, affected dogs attract other males, show reduced libido and/or may urinate in a female posture [14,32,46]. The condition may coexist with contralateral testicular atrophy, infertility, and decreased sperm count (Figure 4).

In some cases, chronic estrogen exposure may lead to bone marrow suppression and pancytopenia. Approximately 10–20% of dogs with feminizing SCTs develop blood dyscrasia, making estrogen-induced myelotoxicity an important and well-recognized paraneoplastic complication rather than a rare event [3,14,16]. Initially, there is transient granulocytic hyperplasia followed by progressive hypoplasia of erythroid, myeloid, and megakaryocytic lineages, ultimately leading to pancytopenia [16]. Clinically, affected dogs may present with lethargy, pallor, petechiae, epistaxis, melena and fever. Hematologic analysis reveals non-regenerative anemia, thrombocytopenia, and leukopenia. Bone marrow aspirates demonstrate marked hypo-cellularity with maturation arrest at early progenitor stages [16]. Pathogenesis involves both direct toxic effects of estrogens on stem cells and vascular damage within the marrow micro-environment, reducing hematopoietic support. These changes are often irreversible or only partially responsive to therapy.

### 3.3. Associated Reproductive Conditions

Several secondary lesions within the reproductive tract can accompany testicular tumors, reflecting the hormonal imbalance produced by the tumor. Squamous metaplasia of the prostate (Paepe et al. 2016) [47] and preputial mucosa occurs secondary to chronic estrogen exposure, most frequently associated with estrogen-secreting SCT [21]. Clinically affected dogs may present with penile/preputial bleeding, and rarely with tenesmus, dysuria, hematuria or serious complications associated with BPH, prostatitis, prostatic cysts and prostatic abscessation [14,47].

Infertility may also be a consequence of testicular tumors. Its mechanism includes tumor-induced degeneration of seminiferous epithelium, breakdown of the blood–testis barrier, HPG axis dysfunction, thermal damage in cryptorchid testes, and autoimmune or inflammatory responses within the gonads [3,9,11]. For these reasons, testicular tumors should be included among the differential diagnoses in breeding males presenting with fertility issues.

## 4. Diagnostic Workflow

**Figure 5:**
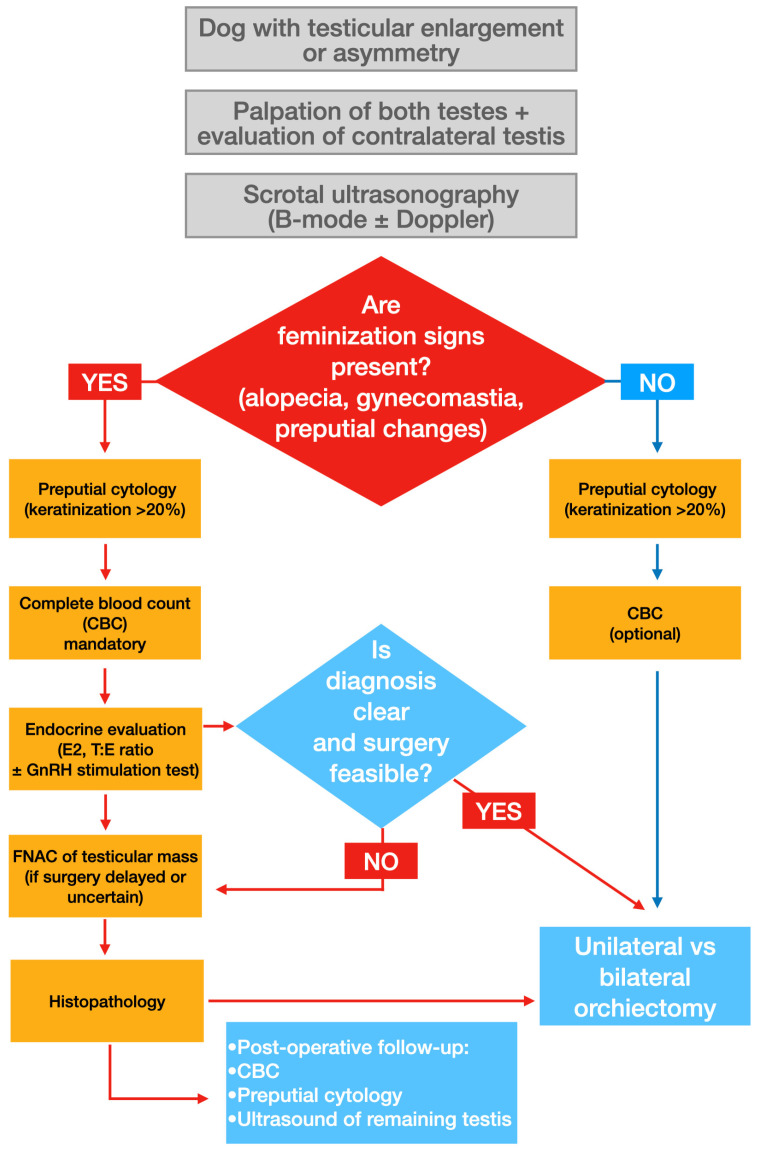
Clinical decision workflow for the diagnosis and management of canine testicular tumors. Color coding: red indicates clinical conditions and decision outcomes; orange represents diagnostic procedures; and blue indicates clinical management and decision steps. Diamond shapes represent decision points within the workflow.

Diagnosis of canine testicular tumors requires integrating clinical examination with imaging and endocrine assessment. Despite the low frequency of metastatic disease, cytological or histopathological confirmation and tumor staging should be performed where possible to ensure appropriate ancillary treatment if indicated (Figure 5). It is important for clinicians to not only investigate reproductive disease in older dogs but remain cognizant of concomitant comorbidities common in old dogs that may be present and mitigate them.

### 4.1. Imaging Techniques

B-mode ultrasonography is the primary imaging tool for evaluating testicular tumors and is generally sufficient to confirm intratesticular lesions. If available, Doppler may be used to assess vascularization and improve lesion characterization [38]. Ultrasonography characterizes the presence, location, vascularity, and architecture of a lesion, but cannot determine tumor type, and malignancy can, at best, be suspected. Computed tomography (CT) is mainly recommended in cryptorchid dogs, especially when the retained testis is intra-abdominal, or if metastatic spread to regional lymph nodes is suspected [48]. Abdominal X-ray has minimal diagnostic value as it rarely identifies retained testes that are small or non-mineralized and cannot reliably assess lymph nodes or identify neoplastic testes [23].

### 4.2. Endocrine and Hormonal Evaluation

Endocrine testing is primarily useful to confirm the presence of hormonally active testicular tumors, especially when presenting with feminization, alopecia or bone marrow suppression (see Supplementary Table S1). In contrast, routine basal hormone measurement alone is of limited diagnostic value and should not be used in isolation. This is because baseline serum testosterone concentration is not a reliable indicator of endocrine activity in dogs with testicular tumors. Furthermore, intact dogs may exhibit testosterone concentrations below <1.0 ng/mL due to physiologic pulsatility and assay variability, and cryptorchidism does not necessarily reduce testosterone production despite impaired spermatogenesis [18]. Thus, resting testosterone values in the range of 0.20–0.40 ng/mL are not clinically meaningful and cannot be used to diagnose cryptorchidism, classify tumor type, or infer endocrine activity. The normal testosterone concentration in healthy adult male dogs is 0.6–9.0 ng/mL [49,50,51].

Assessment of testosterone secretion is only clinically interpretable when combined with a GnRH stimulation test, which causes a temporary increase in Leydig cell function by activating the pituitary–gonadal axis function. A normal testis induces a measurable post-GnRH testosterone rise [52], whereas dogs with estrogen-mediated suppression of the HPG axis or extensive neoplastic replacement may show little or no response [18]. This distinction is clinically useful when testosterone output appears inappropriately low relative to the dog’s clinical status. For feminizing tumors, 17 β-estradiol is the most relevant hormone. In the largest available dataset, dogs with SCT had significantly higher estradiol concentrations (median of 29 pg/mL) and markedly reduced T:E ratios compared to healthy controls, while maintaining testosterone within low-normal ranges [18]. Importantly, clinical feminization is more strongly associated with a reduced T:E ratio than with absolute estradiol elevation, and some SCT dogs with overt feminization have estradiol values within reference limits. Some SCTs lack full estrogen-converting enzymatic activity and therefore produce predominantly estrogenic precursors (e.g., estrone or estrone sulfate) rather than estradiol-17B, meaning that estradiol may remain within the reference range despite marked feminization. Clinicians should therefore prioritize assessing preputial cytology as estrogen’s effects on preputial mucosa are longer lasting and not influenced by pulsatility of hormonal secretion. Normal serum estradiol concentrations in intact male dogs typically range from approximately 5 to 25 pg/mL; however, values should be interpreted cautiously and in conjunction with clinical findings, as low or even reference-range estrogen concentrations do not exclude biologically significant hyperestrogenism [18].

In dogs with ICTs and SEMs, estradiol and testosterone levels did not differ significantly from those of healthy dogs, and feminization was observed in only 1 out of 18 dogs with ICTs and in 0 of 12 dogs with seminomas [18]. These findings reinforce that most endocrine syndromes observed in clinical practice originate from SCTs, particularly when testicles are retained and especially if intra-abdominal [43].

In dogs with uncertain reproductive status, such as suspected prior castration or ambiguous cryptorchid history, anti-Müllerian hormone (AMH) determination represents the most reliable indicator of the presence of functional gonadal tissue, whereas gonadotropin assays (LH, FSH) have limited clinical applicability due to pulsatile secretion and low diagnostic robustness [53,54]. In addition, elevated AMH concentrations can raise a suspicion of a SCT, as neoplastic Sertoli cells often increase AMH production; six dogs with SCT or mixed tumors containing SCTs had AMH concentration exceeding 22 ng/mL, which was significantly higher than the concentration in the control group [55]. Similarly, in another study, serum AMH concentrations in dogs with SCTs were reported to be >22 ng/mL compared with <10 ng/mL in control dogs [56]. These tests have limited relevance once a diagnosis of testicular tumor is already confirmed but remain helpful when a SCT is suspected prior to surgery.

### 4.3. Preputial Cytology

**Figure 6:**
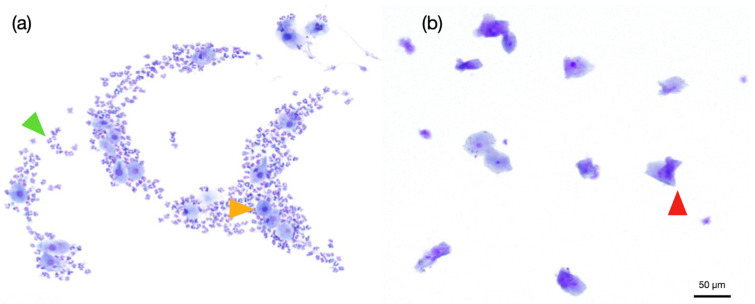
Preputial smear from a normal dog (**a**) and an estrogen-secreting interstitial (Leydig) cell tumor (**b**). Green arrow: neutrophils (PMNs); yellow arrow: intermediate cells; red arrow: keratinized superficial cell; with a 100× magnification.

The preputial epithelium responds to hormonal changes analogously to the vaginal mucosa due to its common embryologic origin [20]. In normal dogs, preputial smears are characterized by intermediate epithelial cells, low keratinization, moderate to high neutrophil presence and high cellularity (Figure 6b) [57]. Preputial cytology is a valuable adjunct tool for identifying estrogen-producing SCTs. A diagnostic threshold of >20% keratinized epithelial cells provides high sensitivity (80%) and specificity (98%) for detecting estrogenic activity in male dogs [21]. In estrogen-exposed males, cytology shows marked keratinization with high proportions of superficial cells, moderate to low numbers of intermediate and parabasal cells, and low neutrophil counts (Figure 6a) [19].

Once the estrogenic source is removed, keratinization gradually decreases, and preputial cytological patterns slowly return to normal. In the Dreimanis study (2012) [21], cytological resolution required 4–8 weeks in most dogs, with some needing up to 12 weeks to achieve full normalization. Clinical signs such as alopecia, hyperpigmentation, gynecomastia, or behavioral feminization often resolve earlier than cytological changes (authors’ unpublished data). This delayed cytological recovery likely reflects the slow turnover of the preputial epithelium and prolonged peripheral effects of estrogenic metabolites.

### 4.4. Fine-Needle Aspiration Cytology

Fine-needle aspiration cytology (FNAC) represents a valuable, minimally invasive diagnostic tool that can often confirm diagnosis of the type of tumor before surgery. Fine-needle aspiration cytology may be considered when surgery is not immediately performed, for example, in older dogs with significant comorbidities, in case surgery must be delayed, or when imaging findings are ambiguous and inflammation cannot be confidently excluded [4,10,21]. Experimental evidence in dogs suggests that fine-needle aspiration causes limited transient local changes, such as mild intratesticular hemorrhage, without clinically relevant long-term damage under the studied conditions [58,59].

Clinical evidence demonstrates its high diagnostic accuracy. In a series of 92 dogs, cytological interpretation showed close agreement with histopathology, with sensitivity of 95% for SEM, 88% for SCT and 96% for ICT, and a specificity of 100% for all tumor types [60]. Distinctive cytological markers such as lymphocytic infiltrates and macro-nucleoli in SEMs, palisading columnar cells in SCTs, and perivascular micro-vacuolated cells with nuclear pseudo-inclusions in ICTs facilitate accurate tumor classification.

### 4.5. Immunohistochemistry and Proliferation Markers

IHC enhances tumor classification and provides prognostic insights, particularly when morphology is ambiguous. SCTs typically express inhibin-α and vimentin, while ICTs show inhibin-α and Melan-A. These markers are particularly helpful in atypical, mixed or morphologically discordant lesions, where routine histology alone may not allow confident classification. SEMs consistently express germ cell markers such as placental alkaline phosphatase (PLAP), c-Kit (CD117), and OCT3/4, and may show variable cytokeratin reactivity depending on the degree of intra-tubular versus diffuse growth [10,12].

Although most SEMs are hormonally inactive, rare androgen-producing cases have been reported. There is no specific IHC marker that reliably identifies androgen-secreting seminomas; instead, diagnosis relies on a combination of tumor classification (via PLAP, c-Kit and OCT3/4), correlation with serum androgen levels, and resolution of clinical signs after orchiectomy [11,18]. In such cases, IHC can help confirm the tumor type, ruling out other androgen-producing neoplasms such as ICTs.

The Ki-67 index serves as a quantitative indicator of proliferative potential, distinguishing indolent from aggressive variants, particularly of SCTs and SEMs, and less commonly ICTs [30]. A minimal IHC panel (inhibin-α, Melan-A, vimentin ± cytokeratin) in addition to Ki-67 quantification resolves most diagnostic ambiguities and provides a pragmatic estimate of proliferative risk in mixed or atypical canine testicular neoplasms where morphology alone is inconclusive [11,12].

### 4.6. Differential Diagnoses

An accurate differentiation between testicular tumors and other scrotal disorders requires integration of clinical findings, ultrasonography, endocrine testing, and, when indicated, FNAC. Several reproductive conditions can mimic testicular tumors, including orchitis, epididymitis, sperm granulomas, testicular torsion, scrotal hernia and segmental aplasia of the epididymis or testis. Inflammatory conditions such as orchitis and epididymitis are typically painful, often characterized by diffuse echogenicity changes and increased vascularity on ultrasonography. Sperm granulomas typically present as firm, well-defined, non-painful nodules; however, rupture of obstructed epididymal ducts with secondary leakage of spermatozoa into the surrounding tissue has been reported in dogs, occasionally resulting in acute inflammatory episodes and pain [11,43]. Testicular torsion presents as sudden hemi-scrotal enlargement, which may or may not be characterized by pain, while scrotal hernia is a slow progressive non-painful scrotal enlargement. Segmental aplasia of the epididymis or testis may present as non-painful unilateral enlargement, tubular dilatation, or cyst-like structures, which remain stable over time, are hormonally inactive and typically do not distort testicular architecture [38]. Ultrasonography remains the cornerstone for discriminating among differential diagnoses.

## 5. Therapeutic Management

Therapeutic management of canine testicular tumors relies primarily on removing the neoplastic testicle through orchiectomy. When the tumor involves only one testicle, unilateral orchiectomy is curative. However, bilateral tumors have been reported in approximately 6–15% of cases, most often synchronously at the time of diagnosis [2,3]. Although true metachronous contralateral primary tumors are uncommon, they have been described, typically developing within 1–3 years following the initial diagnosis. Therefore, periodic clinical examination of the remaining testis is recommended, particularly during the first several years after surgery [11].

### 5.1. Surgical Treatment

Orchiectomy is the treatment of choice for virtually all types of testicular tumors [10,61]. The decision between unilateral and bilateral orchiectomy should be guided by tumor location, endocrine status, age, cytological/histological features when available, and long-term health considerations, as well as the reproductive value of the patient. In addition, retaining gonadal hormones in working dogs is of substantive benefit [62,63,64]. Unilateral orchiectomy could be considered when a single, well-circumscribed intratesticular lesion is present, the contralateral testis is ultrasonographically normal, and the endocrine milieu is within normal limits. Preservation of the healthy testis has gained importance due to the growing body of evidence associating gonadectomy in elderly male dogs with increased risks of prostatic carcinoma and obesity [6,17]. Thus, in the absence of contralateral abnormalities, testis sparing through unilateral orchiectomy may be considered and discussed with the owner, making sure that the risk of potential metachronous neoplastic development on the remaining scrotal testicle is clearly communicated. Similarly to primary lesions, secondary neoplastic development typically occurs slowly, which may allow the use of their unilaterally castrated dog for reproductive purposes if desired [61].

Historically, bilateral orchiectomy has been advocated in dogs with feminizing SCTs, particularly when contralateral testicular atrophy is present. However, it is important to stress that hormone secretion itself does not increase the risk of malignancy, invasiveness, or metastatic disease, and no evidence supports the notion that a hormonally active neoplastic testicle causes simultaneous pathological changes of the contralateral testis, thus making complete surgical castration necessary [2,11]. The contralateral testicular atrophy observed in such dogs is largely functional and secondary to HPG axis suppression; it does not indicate neoplastic transformation and may occasionally resolve once the neoplastic testicle is removed, provided that hormonal downregulation has not lasted very long [11,16]. Therefore, bilateral orchiectomy in hormonally active tumors should not be considered routine but rather reserved for specific situations, such as when the owner chooses bilateral gonadectomy for non-reproductive management reasons, or when concurrent androgen-dependent conditions such as perineal gland adenomas or benign prostatic hyperplasia justify removal of androgen sources [61]. Lastly, risk–benefit assessment of old dogs with severe comorbidities such as renal or cardiopulmonary compromise may present considerable anesthetic and surgical risks. In such cases, where there is no evidence of hormone secretion or serious prostatic disease, the lesser risk might be to keep monitoring the patient and not to perform orchiectomy of either testicle.

### 5.2. Adjuvant Therapy

Management of hyperestrogenism-induced bone marrow aplasia is largely supportive and centers on keeping the patient stable while bone marrow function recovers following removal of the estrogen source. This recovery may be absent, partial or complete depending on severity of bone marrow suppression, duration and individual case. The estrogen-related myelotoxicity is a well-recognized paraneoplastic effect of functional SCTs and remains a clinically challenging condition, with recovery that can be slow, unpredictable, and sometimes incomplete despite removal of the offending testicle and its tumor [11,65]. The intensity of supportive care depends on the degree of cytopenia. Dogs with clinically relevant anemia often require transfusion with packed red blood cells or whole blood, while severe thrombocytopenia or active bleeding may require platelet concentrates [33,66]. In neutropenic patients, early and aggressive broad spectrum antimicrobial therapy is essential to reduce the risk of sepsis, particularly during the immediate postoperative period when bone marrow function has not yet recovered [65]. Throughout this phase, fluid therapy and attentive nursing care play a key role in maintaining systemic stability.

Attempts to pharmacologically stimulate hematopoiesis have produced mixed and often inconsistent results. Hematopoietic growth factors such as granulocyte colony stimulating factor and erythropoietin have been used empirically in selected cases, but controlled data in dogs are lacking, and bone marrow recovery has been documented in some patients following tumor removal alone, without the use of stimulatory agents [65]. Lithium carbonate (0.5–1.8 mmol/L) has been reported as a potential adjunct in dogs with estrogen-induced pancytopenia, including refractory cases, yet its narrow therapeutic margin and risk of nephrotoxicity require careful monitoring and limit its routine clinical use [66,67]. In a small number of refractory cases, a short course of glucocorticoids may provide a temporary increase in hematopoietic output; however, this must be weighed cautiously against the risk of further immunosuppression in already vulnerable patients [16].

### 5.3. Staging and Follow-Up

Because endocrine abnormalities and systemic complications occur almost exclusively with estrogen-producing SCTs, follow-up requirements for these tumors are substantially different from those with hormonally inactive tumors. In dogs with a testicular mass, if preputial cytology is unremarkable and feminization signs are not present, castration may be postponed or avoided depending on the patient’s age and general health conditions, systemic risk, and owner’s preference. Inguinal lymph node palpation as well as ultrasound evaluation of the medial iliac lymph nodes should always be performed to identify potential regional metastases [61]. Hormonally active tumors are a concern when producing estrogens; in these subjects, keratinization of preputial cytology is thought to be a precocious sign of hyperestrogenism followed by clinical signs of feminization and/or bone marrow dysplasia [14,16]. Whenever clinical or even only preputial cytology signs of estrogen production are present, surgical removal of the estrogen-secreting neoplastic testicle should be seriously considered. Preputial cytology should be performed in all cases of dogs with a suspected testicular tumor and, in dogs without signs of estrogen secretion on the first clinical exam [19,21], repeated every 6 months to rule out estrogen secretion in case of new neoplastic development. FNAC remains the only minimally invasive method capable of providing a presumptive diagnosis [59].

In dogs with hematological alterations resulting from functional tumors subjected to bilateral orchiectomy, complete blood counts are recommended weekly during the first two weeks after surgery to monitor bone marrow recovery. In dogs undergoing unilateral orchiectomy, preputial cytology (and T:E ratio) should be reassessed every four weeks to confirm endocrine normalization; at three months, ultrasonographic evaluation of the contralateral testis and regional lymph nodes is recommended for all dogs undergoing unilateral orchiectomy; further imaging of the remaining testis should be performed at six and twelve months, and annually for at least two years thereafter, to detect delayed contralateral disease or late recurrence [11,47].

## 6. Prognosis

The prognosis of testicular tumors varies according to tumor type, hormonal secretion, and the presence or absence of systematic estrogenic effects (Table 1) and the presence of prostatic disease. Most canine testicular tumors are benign and have an excellent outcome following complete surgical excision when detected early [3,10,11].

SCTs generally carry a good prognosis after removal, particularly when confined to the testis [3]. Only approximately 25–30% of SCTs are hormonally active [10,11]. Therefore, the major prognostic determinant is not the tumor itself but the reversibility of estrogen-induced myelotoxicity [14,16]. Dogs with reversible cytopenia typically recover hematologically within 4–8 weeks [14,16], whereas irreversible bone marrow aplasia is associated with a guarded prognosis and may lead to early mortality despite orchiectomy [14,39]. In dogs with estrogen-secreting SCTs, clinical signs of feminization may regress after surgery, with marked improvement after a month and complete recovery within approximately three months [34]. Interestingly, SCTs have been reported as a primary lesion in an extra-testicular location in 12 castrated dogs several years following castration, with feminization signs developing in two dogs and disappearing after tumor removal [68,69].

ICTs are almost always benign, and prognosis is excellent after orchiectomy [3,10]. Metastatic ICTs are exceptionally rare. Although these tumors are usually hormonally inactive, occasional hormonally active cases exist that produce low-level androgenic or estrogenic activity, but these cases are do typically not impact prognosis [18,19]. Similar to SCTs, one case of an ICT was diagnosed as a primary, extra-testicular lesion in an elderly dog who was gonadectomized at a young age [69].

SEMs are typically non-metastatic and carry a very favorable prognosis following surgical excision [4,5]. Nevertheless, malignant SEMs with pulmonary [70], lymph node and bone metastasis [71] have been reported. A small number of cases of disseminated SEMs with visceral metastases involving the lungs, liver, spleen, kidneys and adrenal glands have been reported [72,73,74]. Although a small minority of testicular tumors may behave more aggressively with vascular invasion or nodal metastasis, overall long-term survival is normally good [30,38].

Cryptorchid testicles with tumors (SCTs or SEMs) carry a slightly higher risk due to delayed detection, tend to be larger at diagnosis, and are more frequently associated with estrogenic systemic effects, reflecting the higher proportion of functional SCTs among cryptorchid testes. Nevertheless, the prognosis remains good when tumors are diagnosed prior to the development of severe bone marrow toxicity [9,14,28].

In breeding males with hormonally inactive tumors, unilateral orchiectomy may preserve fertility. Postoperative semen evaluation is recommended only when owners intend to continue breeding. Hormone-secreting tumors generally impair fertility through endocrine suppression, and recovery of spermatogenesis after surgery depends on the duration of exposure and contralateral testicular integrity [75].

**Table 1:** Summary of tumor type, hormonal activity, metastatic potential, systemic effect, and postoperative prognosis.

Tumor Type	Hormonal Secretion	Metastatic Potential %	Systemic Effects	Prognosis After Surgery
SCT	Often (25–30%) estrogen-secreting [7,10,14,16,21,24,34]	≤15% [10,65,76]	Feminization, myelotoxicity [14,16,40,65,66]	Good (if early), guarded (if marrow aplasia)
ICT	Very Low [41,42]	<1% [10,12,13,42,43]	Rare [41,42]	Excellent
SEM	Very Low [5,10,70]	≤5–10% [5,10,12,70,71,72,73,74,77]	Minimal/None [70]	Excellent

## 7. Conclusions and Future Perspectives

Early detection and orchiectomy cure most testicular tumors. However, endocrine complications such as hyperestrogenism and associated myelotoxicity are potentially fatal complications. A combined diagnostic approach integrating clinical assessment, ultrasound, preputial cytology, and T:E ratio supports early detection and informed surgical decision making. Prostatic health permitting, unilateral orchiectomy may be discussed with the owners, both in hormonally active and inactive tumors when only one testis is involved. Allowing the healthy gonad to be spared should be reserved for select cases where the breeding potential is to be retained or working ability is still a priority but should be followed by regular reproductive health monitoring. Future research should clarify prognostic indicators and validate immunohistochemical markers to improve risk stratification and long-term management.

## Data Availability

No new data were created or analyzed in this study. Data sharing is not applicable to this article.

## Supplementary Materials

The following supporting information can be downloaded at: https://www.mdpi.com/article/10.3390/ani16081202/s1, Figure S1: Normal histological architecture of the canine testis, showing well-organized seminiferous tubules with an intact spermatogenic series and a clearly defined lumen. Figure S2: Histological features of a Sertoli cell tumor, with neoplastic cells arranged in tubules, cords, and islands, supported by fibrous stroma and showing cytoplasmic vacuolization. Figure S3: Histological appearance of an interstitial (Leydig) cell tumor, composed of large polygonal cells with eosinophilic granular cytoplasm and occasional cytoplasmic vacuolation. Figure S4: Histological appearance of seminoma, consisting of uniform large round to polygonal neoplastic cells with evident mitotic figures and cellular pleomorphism. Table S1: Summary of endocrine diagnostic tests used in the evaluation of canine testicular tumors, including sample type, recommended assay methods, clinical applications, and key limitations.
