# Supplementary material for: Clinical Management of Testicular Tumors in Dogs
Source: Animals (Basel). 2026 Apr 15;16(8):1202. doi: 10.3390/ani16081202 (PMC13113165; doi:10.3390/ani16081202)
Supplement: Supplementary file 1 [file animals-16-01202-s001.zip › supplementary table 1.pdf]

**Table S1.** Summary of endocrine diagnostic tests used in the evaluation of canine testicular tumors, including sample type, recommended assay methods, clinical applications, and key limitations.

| Hormone | Sample Type | Recommended Assay Method | Clinical Use | Key Limitations |
|---|---|---|---|---|
| **Testosterone (T)** [18] | Serum | Validated immunoassay CLIA, RIA, FEIA preferred; ELISA acceptable) | Leydig cell function; GnRH test | Pulsatility; inter-assay variability |
| **17β-estradiol (E2)** [18] | | High-sensitivity immunoassay or LC-MS/MS (preferred if available) | Detect estrogen-producing tumors | Low concentrations poor accuracy with standard ELISA |
| **T:E ratio** [18] | | Derived from validated T and E2 assays | Best indicator of hyperestrogenism | Depends on assay accuracy |
| **AMH** [55] | | Species-specific ELISA (validated kits) | Detect testicular tissue / SCT suspicion | Limited role post-diagnosis |
| **GnRH stimulation test**[51] | | Functional test (T measurement) | Assesses Leydig cell responsiveness when basal T unclear | Requires at least 2 samples |
