# Supplementary figures and images for: Clinical Management of Testicular Tumors in Dogs
Source: Animals (Basel). 2026 Apr 15;16(8):1202. doi: 10.3390/ani16081202 (PMC13113165; doi:10.3390/ani16081202)

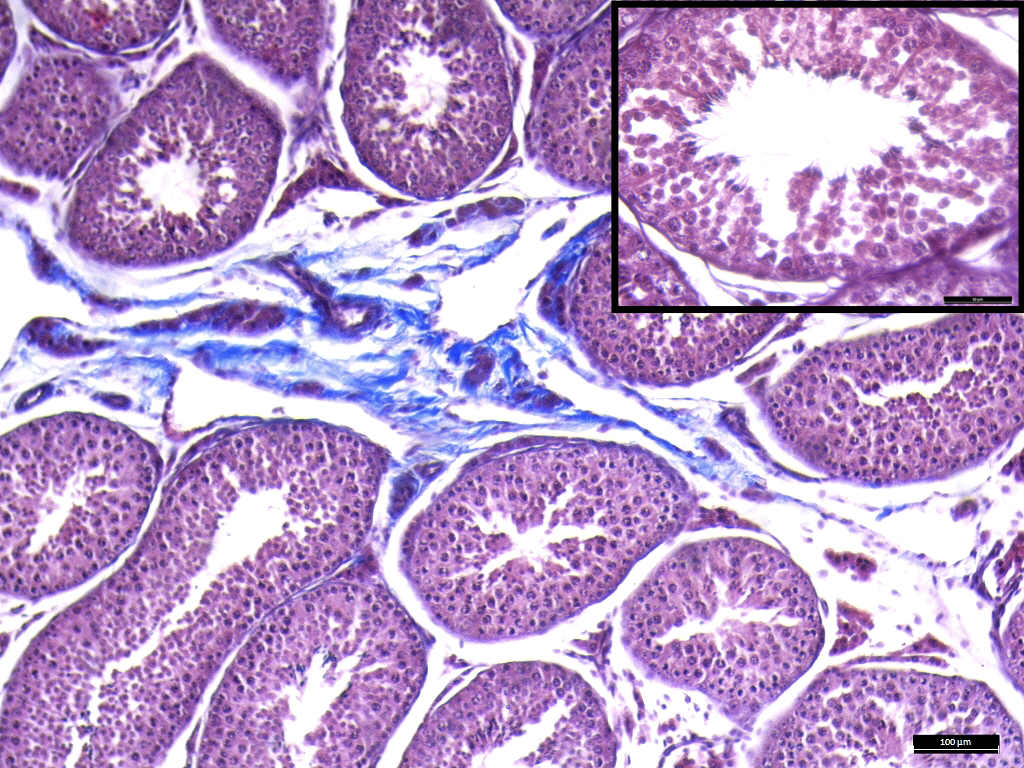

Supplement: Supplementary file 1 [file animals-16-01202-s001.zip › Supp figure 1 Normal histology.001.tiff]

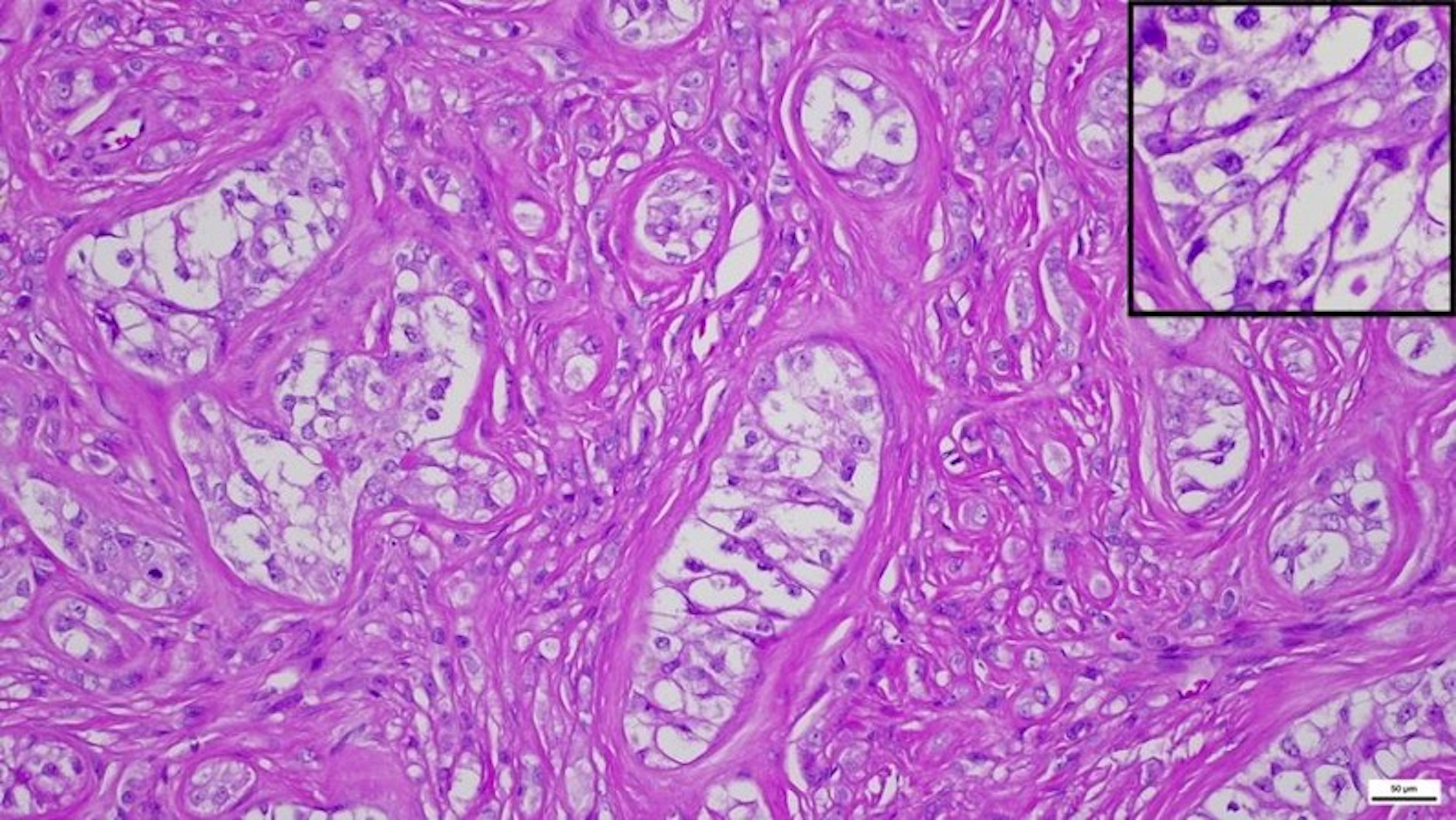

Supplement: Supplementary file 1 [file animals-16-01202-s001.zip › Supp figure 2 SCT histopatology.tiff]

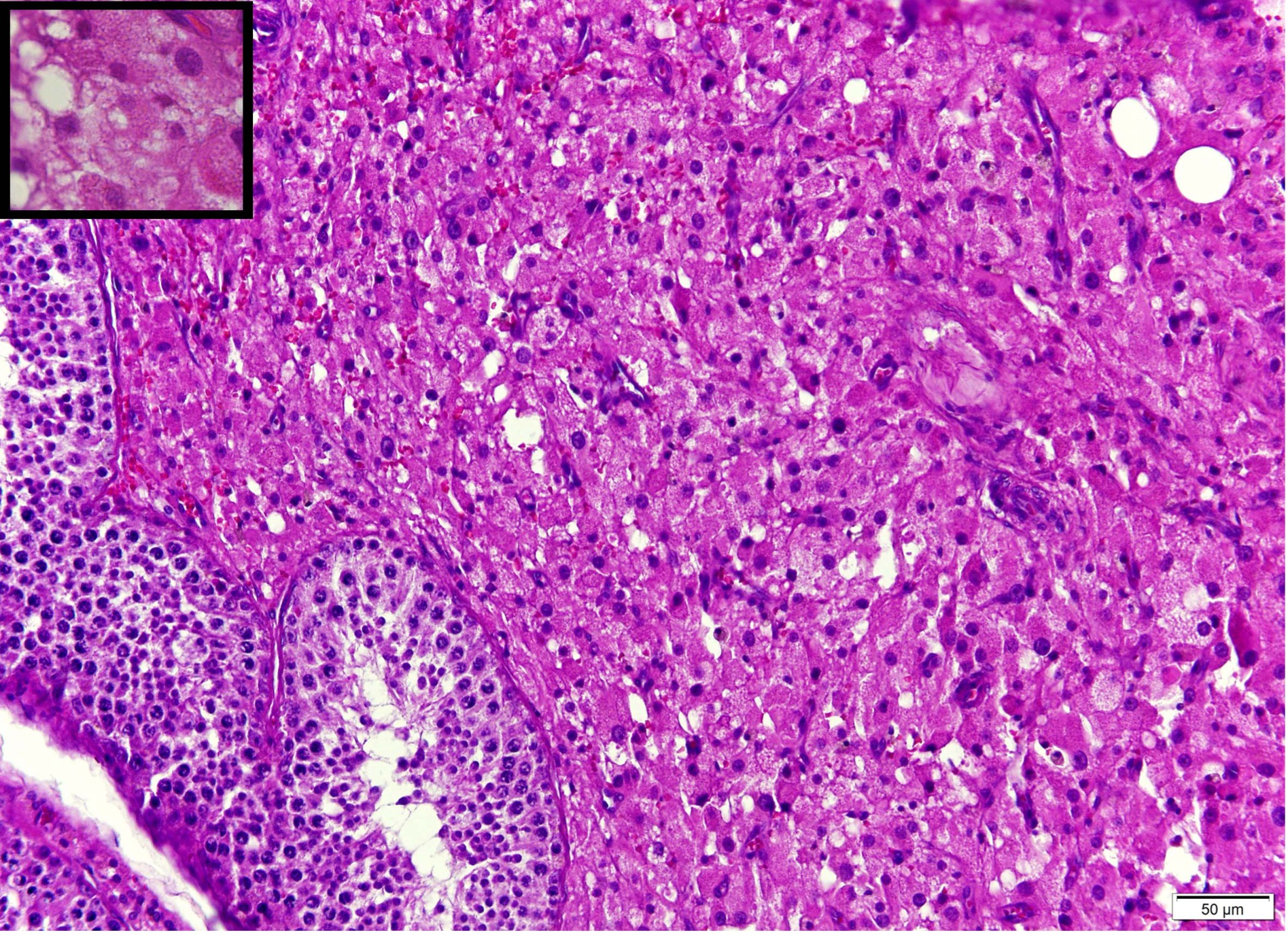

Supplement: Supplementary file 1 [file animals-16-01202-s001.zip › Supp figure 3 ICT histopatology.tiff]

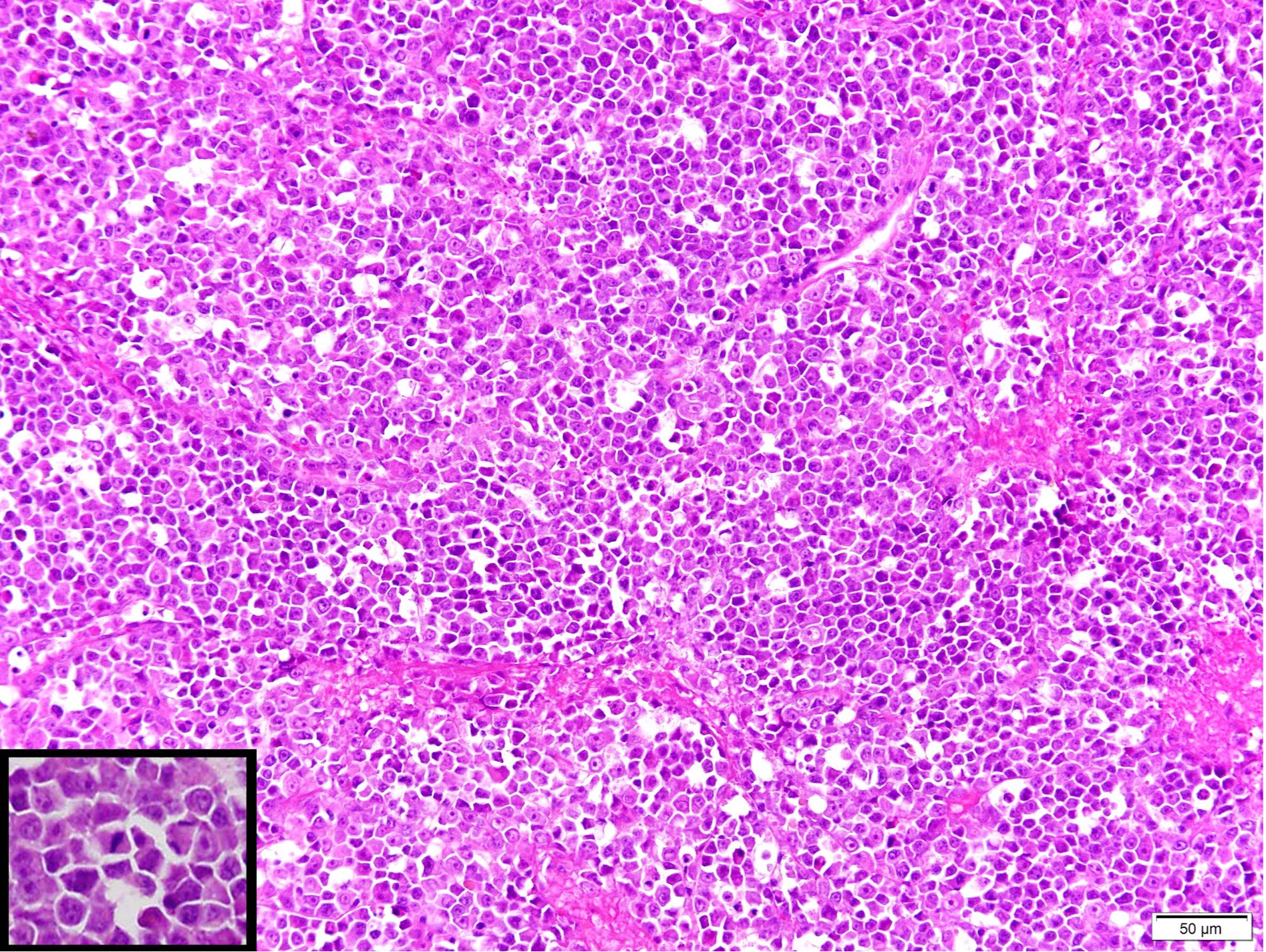

Supplement: Supplementary file 1 [file animals-16-01202-s001.zip › Supp figure 4 SEm histopatology.tiff]
